# C1 inhibitor attenuates pulmonary inflammation in an *in vivo *model of transfusion-related acute lung injury

**DOI:** 10.1186/cc12310

**Published:** 2013-03-19

**Authors:** M Müller, PR Tuinman, G Jongsma, AM Tuip-de Boer, L Boon, SS Zeerleder, NP Juff ermans

**Affiliations:** 1 Academic Medical Center, Amsterdam, the Netherlands; 2 Bioceros, Utrecht, the Netherlands

## Introduction

Transfusion-related acute lung injury (TRALI) has a high incidence in critically ill and surgical patients and contributes to adverse outcome, while specific therapy is absent. Recently it was demonstrated that complement activation plays a pivotal role in TRALI. We aimed to determine whether a C1 inhibitor is beneficial in a two-hit mouse model of antibody-mediated TRALI.

## Methods

BALB/c mice were primed with lipopolysaccharide (LPS, from *E. coli *0111:B4) that was administered intraperitoneally in a dose of 0.1 mg/kg, after which TRALI was induced by injecting MHC-I antibody against H2Kd (IgG2a,k) at a dose of 2 mg/kg. Mice infused with PBS or LPS served as controls. Concomitantly, mice infused with the MHC-I antibody were treated with C1 inhibitor (Cetor^®^; Sanquin, Amsterdam, the Netherlands) in a dose of 400 IU/kg intravenously. After infusion, mice were mechanically ventilated with a lung-protective pressure-controlled mode for 2 hours and then sacrificed, after which a bronchoalveolar lavage (BAL) was done. Statistics were analyzed by one-way ANOVA, values expressed as mean and standard deviation.

## Results

Injection of LPS and MHC-I antibodies resulted in TRALI, indicated by increased levels of protein in the BAL fluid, wet/dry ratios and levels of KC, MIP-2 and IL-6. C1 inhibitor Cetor^® ^significantly reduced total protein in BAL fluid from 792 (169) to 421 (230) μg/ml (*P <*0.001) and tended to reduce the wet/dry ratio from 5.3 ± 0.3 to 4.9 ± 0.5 (*P *= 0.09). Cetor^® ^also reduced BALF levels of MIP-2 from 214 (54) to 127 (22) pg/ml (*P <*0.01). KC and IL-6 levels were not affected.

## Conclusion

In a model of antibody-mediated TRALI, C1 inhibitor attenuated pulmonary inflammation. C1 inhibition may be a potential beneficial intervention in TRALI.

